# Giant congenital diaphragmatic hernia in an adult

**DOI:** 10.1186/1749-8090-9-31

**Published:** 2014-02-10

**Authors:** Yubin Zhou, Heng Du, Guowei Che

**Affiliations:** 1 Department of Thoracic Surgery, West-China Hospital, Sichuan University, Chengdu 610041, Sichuan, Province, PR China

**Keywords:** Congenital diaphragmatic hernia, Bochdalek hernia, Diaphragmatic eventration

## Abstract

Bochdalek hernia is the most common type of congenital diaphragmatic hernia. It appears frequently in infants but rarely in adults. We present the case of a 50-year-old female han patient with tremendous left-sided congenital posterolateral diaphragmatic hernia (Bochdalek hernia) who also has a pair of supernumerary breasts and pulmonary hypoplasia of the lower-left lobe. The patient had an experience of misdiagnosis and she was treated for bronchitis for one year until being admitted to our hospital. This case study emphasizes the rare presentation of Bochdalek hernia in adults and the necessity of high clinical attention to similar cases.

## Background

The estimated rate of occurrence of congenital diaphragmatic hernia varies from 1:2000 to 1:4000 live births. Though thousands of such cases are reported during infancy, few reports describe adult patients diagnosed with Bochdalek hernia. Usually, these hernias become evident during the neonatal period, through symptoms and signs of respiratory distress. However the most frequent symptoms found in adult patients are slight respiratory and digestive symptoms. It is also possible for these hernias to be asymptomatic. Here we report a case of mild dyspnea within a congenital posterolateral diaphragmatic hernia.

## Case presentation

**Figure 1:**
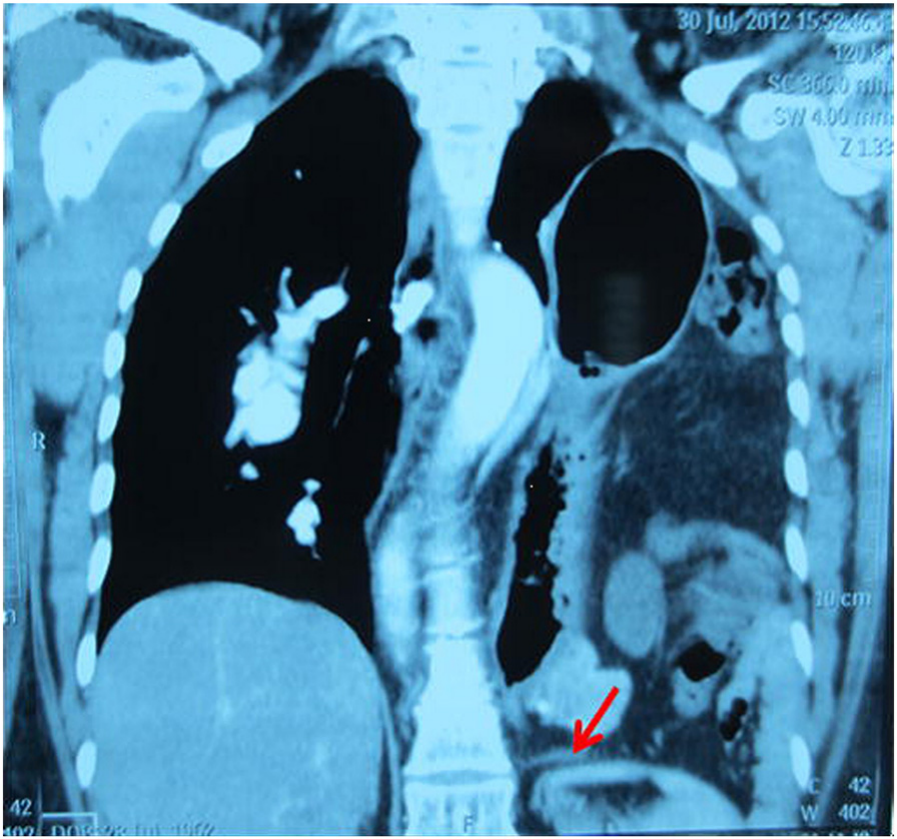
MPRs showed the left thoracic cavity was completely filled with abdominal organs reaching to the apex of the pleura cavity and the interrupted hemidiaphragm of Bochdalek hernia (the arrowhead).

**Figure 2:**
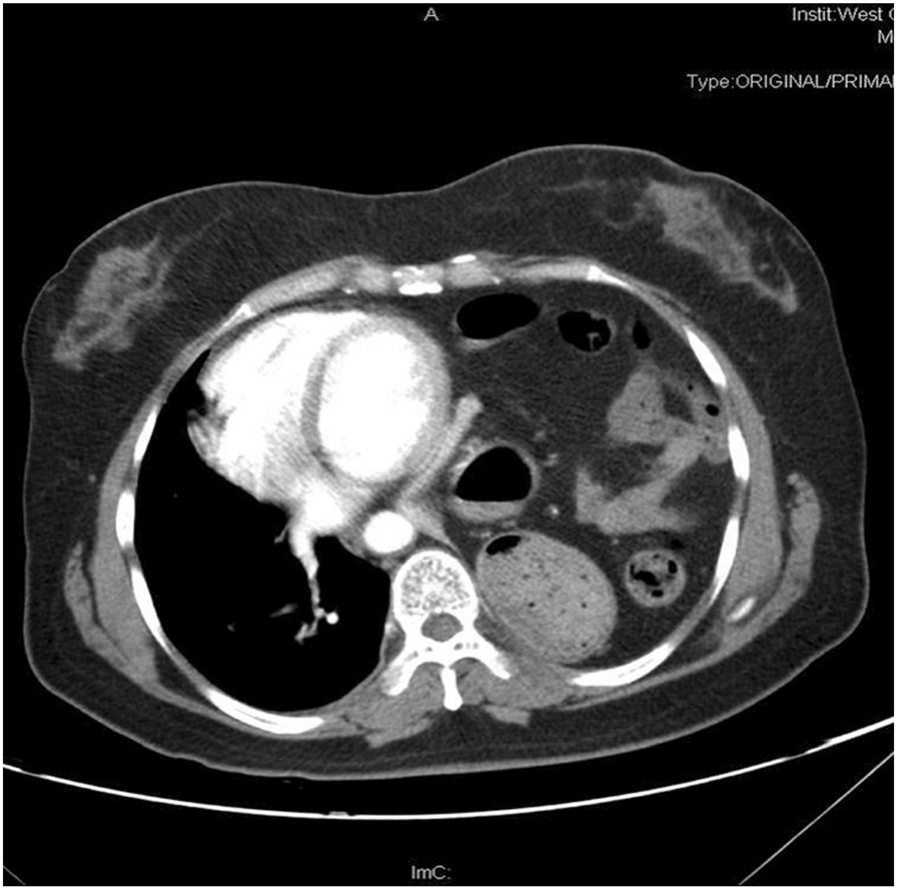
Contrast-enhanced CT scans showed the mediastinum shifted to the right thoracic cavity.

**Figure 3:**
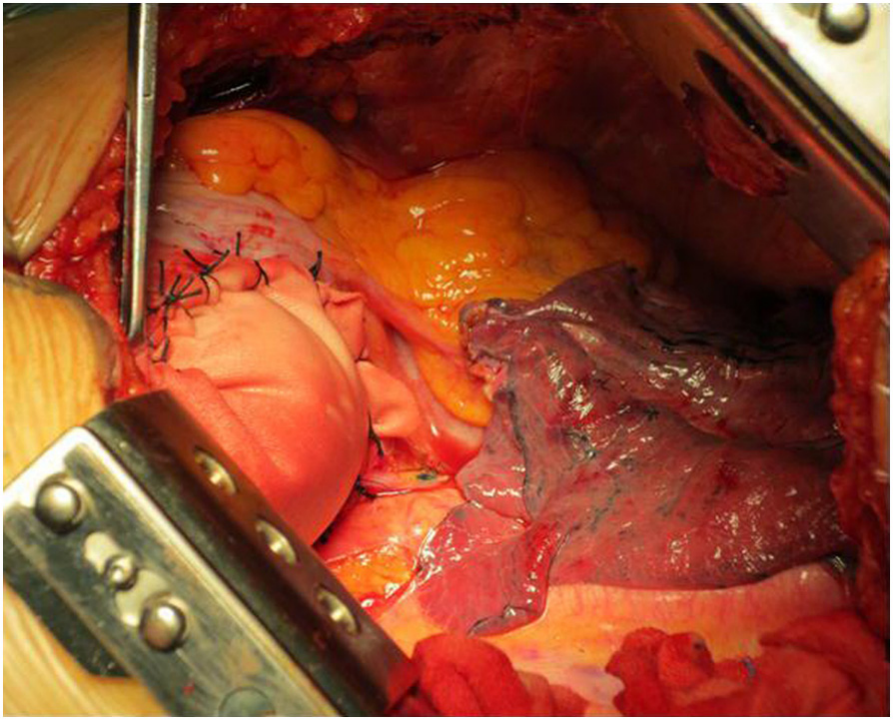
Intraoperative picture showed the posterolateral defect repaired by Dacron patch and the hypogenetic left lower lobe.

**Figure 4:**
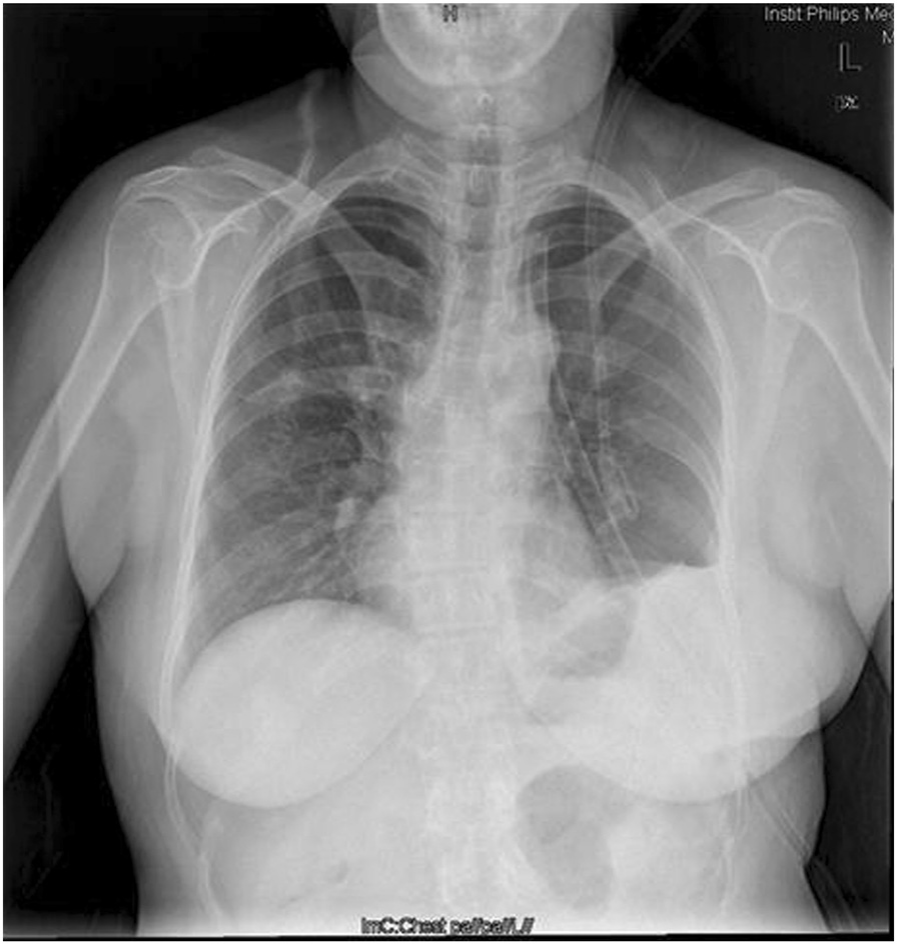
The repeated chest radiograph after 4 days of surgery showed the well recruitment of the left lung.

A 50-year-old female patient of Han nationality was admitted to our department with a one year history of slight dyspnea. The discomfort was greatly obvious after heavy physical work, but could be alleviated by taking a rest. She denied having other symptoms such as chest pain, abdominal pain, dysphagia, vomiting, and dyspepsia. There was no history of trauma or surgery. Upon physical examination we inspected nothing characteristic except the remarkable decrease of breathing sounds and increased bowel sounds in the left hemithorax. Contrast-enhanced chest computed tomography(CT) and multiplanar reconstructions(MPRs) showed a notable displacement of the abdominal viscera into the left thoracic cavity and the interrupted hemidiaphragm [Figure 1]. The atelectasis of the left lung was serious for most of the whole left thoracic cavity was filled by the atopic abdominal organs. The heart was also extruded into the right hemithorax and the right lung was partially compressed [Figure 2]. As the preoperative diagnosis that is a left-sided diaphragmatic hernia made, the patient was taken in for surgery. Under general anesthesia using a double- lumen tube, a standard posterolateral thoracotomy entering the chest through the seventh intercostal space was made. During the operation, it was found that most of the left thoracic cavity was filled with abdominal organs reaching to the apex of the pleural cavity. When the exploration of the thoracic cavity was performed, we found the stomach, small bowel loops, omentum and a partial transverse colon. There was no remarkable adhesion except around the hernial sac. After reducing the abdominal organs, a defect located on the left posterolateral side of the hemidiaphragm and measuring approximately 10*8 cm became visible. We also found that the left upper and lower lobes were subtotally atelectatic. The defect was primarily repaired by a Dacron patch and was sutured with a non-absorbable monofilament [Figure 3]. The procedure was ended with the left thoracic cavity drained by a single chest tube(28 F). After the surgery we diagnosed the patient had a left-sided Bochdalek hernia. In the post-op period, the patient had an uneventful and smooth recovery. A repeated chest radiograph after 4 days of surgery revealed that the re-expansion of the left lung went very well [Figure 4]. She was discharged home without any respiratory or gastrointestinal symptoms.

## Discussion

Bochdalek hernias, first described by Bochdalek in 1848, are characterized by a congenital defect on the posterolateral region of the diaphragm without hernia sac. They are generally discovered in neonates, but is rarely reported in adults. The aetiology factor of Bochdalek hernias is unknown, but we know that the occurrence of this disease is due to the failure of closure of the pleuroperitoneal canal during the ninth to tenth week of gestation [1]. According to reported literatures, Bochdalek hernias are associated with other congenital anomalies in 25-57% of cases and with chromosomal disorders in 10-20% of cases. In our case, the patient has a pair of supernumerary breasts and the pulmonary hypoplasia of the lower-left lobe. The diagnosis of a Bochdalek hernia in adults is not easy and it is commonly misdiagnosed. Unlike infants who show with respiratory distress early, the most frequent symptom in adults is mild discomfort and 25% of adult patients are asymptomatic. Consequently, many patients are merely treated according to their symptoms. No more diagnostic investigation is pursued due to the lack of awareness of the disease. Our patient also had an experience of misdiagnosis and she was treated for bronchitis for one year until she was admitted to our hospital. Thus it is important for us to keep the disease in mind. When clinical suspicion of Bochdalek hernia is produced, multiple imaging modalities are available. X-rays are the most general imaging study performed to evaluate the diaphragm and thoracic cavity. When chest radiographs are indeterminate, Spiral CT and MPRs are a good choice to offer us more information. The differential diagnosis of a huge mass in the left thoracic cavity is congenital diaphragmatic eventration. Diaphragmatic eventration is characterized by the displacement of all or a part of the intact diaphragm. By contrast, the diaphragm of a patient with a Bochdalek hernia is interrupted and has a defect on it. On the other hand, diaphragmatic eventration does not always need surgical therapy. However for congenital diaphragmatic hernia, surgical repair should be performed as soon as the diagnosis is confirmed in order to avoid serious complications. The principal management of Bochdalek hernias include reducing the abdominal organs and repairing the defect. It is controversial as to which approach is the best. Scholars who choose thoracotomy praise the convenience of separating adhesions between thoracic contents and the hernia sac, although 62% to 90% of Bochdalek hernias do not have hernial sac, however the presence of sac is not the rule [2]. Those who advocate a laparotomy claim that the abdominal approach is better than thoracotomy for dealing with possible complications such as malrotation, obstruction, strangulation and perforation of abdominal viscera [3]. Minimal invasive surgeries including thoracoscopic repair and laparoscopic repair of Bochdalek hernia are also reported [4]. Our patient underwent a thoracotomy in consideration of the presence of adhesions between thoracic contents and pleura.

## Conclusion

For Bochdalek hernias, correct diagnosis and early treatment is significant to avoid the occurrence of serious complications. To improve the quality of medical treatment for Bochdalek hernias in adults, more cases will need to be reported and long-term follow up should proceed.

## Consent

Written informed consent was obtained from the patient for publication of this case report and any accompanying images. A copy of the written consent is available for review by the Editor-in-Chief of this journal.

## Competing interests

All the authors declare that they have no competing interests.

## Authors’ contributions

In the following we specify the individual contributions of authors to the manuscript. All surgical procedures were performed by GWC, YBZ and HD. YBZ and HD managed the perioperative period of the patient. YBZ prepared the manuscript and GWC made final approval. All authors read and approved the final manuscript.

## References

[B1] Pollack LD Hall JG Posterolateral(Bochdalek’s) diaphragmatic hernia in sisters Am J Dis Child 1979 9 1186 1188 507010 10.1001/archpedi.1979.02130110094019

[B2] Almeida CEC Reis LS Almeida CMC Adult right-sided Bochdalek hernia with Ileo-Cecal appendix: Almeida-Reis hernia Int J Sur Case Reports 2013 9 778 781 10.1016/j.ijscr.2013.06.006 PMC3741443 23872263

[B3] Fingerhut A Baillet P Oberlin P More on congenital diaphragmatic hernia in the adult Int Surg 1984 9 182 183 6500888

[B4] Ray U Maity B SenGupta TK Laparoscopic repair of late presenting congenital Bochdalek diaphragmatic hernia J Indian Med Assoc 2011 9 435 436 22315780

